# Supplementary material for: Tumorigenicity decrease in Bcl-xL deficient MDCK cells ensuring the safety for influenza vaccine production
Source: PLoS One. 2024 Dec 16;19(12):e0311069. doi: 10.1371/journal.pone.0311069 (PMC11649150; doi:10.1371/journal.pone.0311069)
Supplement: S1 File — (ZIP) [file pone.0311069.s002.zip › S2_File/4_KOG_enrichment/network/union.DE_gene.subcluster1_KOG_enrichment_network.pdf]

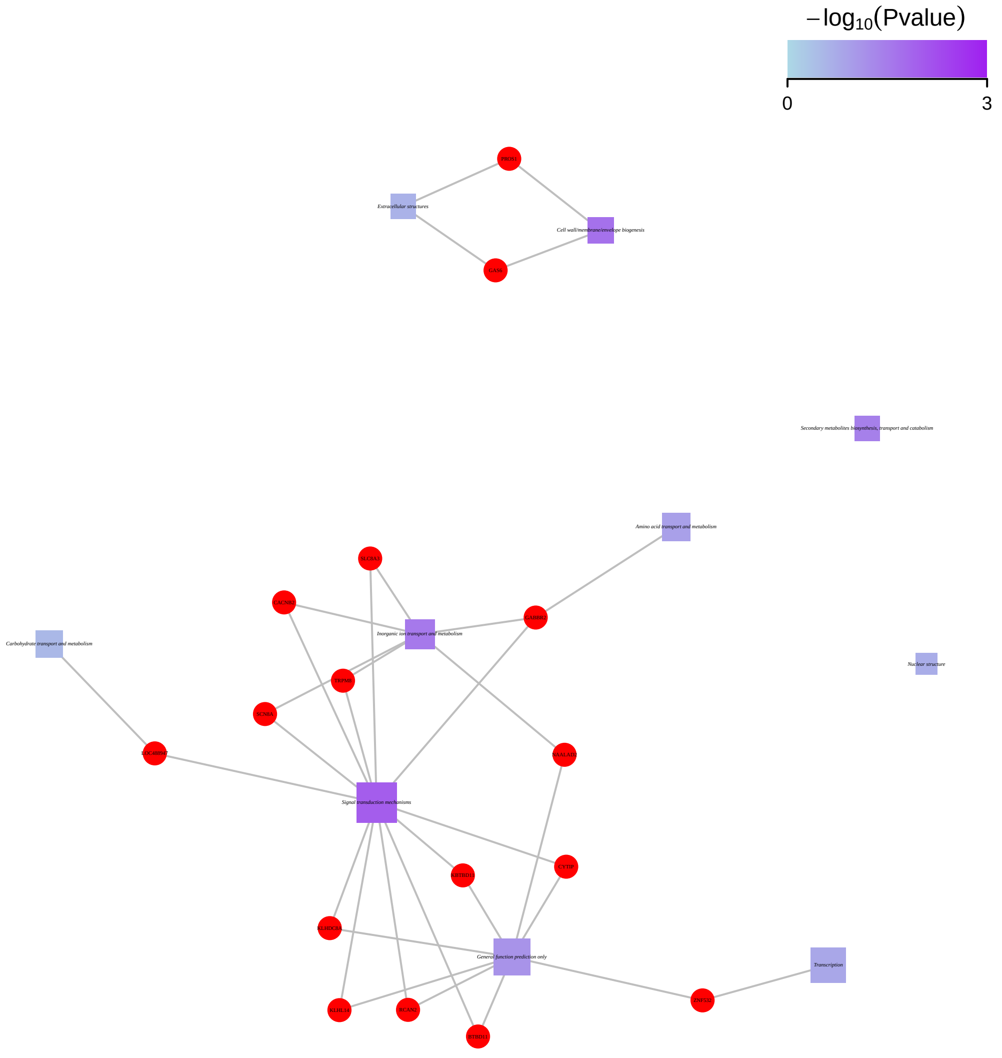

$-\log_{10}(\text{Pvalue})$
0
3
PROS1
Extracellular structures
Cell wall/membrane/envelope biogenesis
GAS6
Secondary metabolites biosynthesis, transport and catabolism
Amino acid transport and metabolism
SLC8A3
CACNB2
GABBR2
Carbohydrate transport and metabolism
Inorganic ion transport and metabolism
Nuclear structure
TRPM8
SCN8A
LOC488947
NAALAD2
Signal transduction mechanisms
KBTBD11
CYTIP
KLHDC8A
General function prediction only
Transcription
ZNF532
KLHL14
RCAN2
BTBD11
